# Evaluation of Serum Amyloid A as a Marker of Persistent Inflammation in Patients with Rheumatoid Arthritis

**DOI:** 10.1155/2015/843152

**Published:** 2015-01-28

**Authors:** Mehmet Agilli, Fevzi Nuri Aydin, Tuncer Cayci, Yasemin Gulcan Kurt

**Affiliations:** ^1^Department of Biochemistry, Agri Military Hospital, 04100 Agri, Turkey; ^2^Department of Biochemistry, Sirnak Military Hospital, 73010 Sirnak, Turkey; ^3^Department of Medical Biochemistry, Gulhane Military Medical Academy, 06018 Ankara, Turkey

We read with great interest the paper by Targońska-Stępniak and Majdan titled “Serum Amyloid A as a Marker of Persistent Inflammation and an Indicator of Cardiovascular and Renal Involvement in Patients with Rheumatoid Arthritis” in which the investigators reported that high serum amyloid A (SAA) concentration was strongly associated with activity of the disease and risk of cardiovascular and renal involvement in patients with rheumatoid arthritis [1]. We thank the authors for their detailed report. However, we wish to make some comments on SAA.

SAA is produced in the liver in response to proinflammatory cytokines such as tumor necrosis factor alpha (TNF-*α*), interleukin-1 (IL-1), and IL-6 [2]. Previous studies suggested that several diseases such as ankylosing spondylitis, autoinflammatory diseases (Hashimoto's thyroiditis, familial Mediterranean fever), major depression, systemic lupus erythematosus, diabetes mellitus, inflammatory bowel diseases, acute pancreatitis, several types of vasculitis, psoriasis, and epilepsy could affect SAA levels [3, 4]. In addition to these diseases, glucocorticoids, statins, disease-modifying antirheumatic drugs, corticosteroids, and nonsteroidal anti-inflammatory drugs could alter SAA levels [5, 6]. Also, dietary food supplements such as antioxidants (ascorbic acid, taurine, and phytic acid), vitamin E, vitamin A, *α* linoleic acid, omega-3 fatty acids, and polyunsaturated fatty acids can influence SAA levels [7, 8]. In this respect, without defining these contributing factors, interpreting the results is problematic.

Alcohol use and smoking status of participants are other confounding factors for SAA measurement [9, 10]. Therefore, these factors have to be expressed and a multivariate regression analysis should be applied to show whether these variables have an impact on SAA levels.

In conclusion, clarifying these concerns will certainly provide a clearer picture when interpreting SAA levels among participants.
